# Superimposed type 2 segmental atopic dermatitis: case series and review of the literature

**DOI:** 10.1111/ced.15196

**Published:** 2022-05-22

**Authors:** Efrat Bar‐Ilan, Jacob Mashiah, Ronen Alkalay, Arieh Ingber, Keren Or Zahavi, Jonathan Bar, Tal Zeeli, Andrea Gat, Liat Samuelov, Eli Sprecher, Ilan Goldberg

**Affiliations:** ^1^ Division of Dermatology Tel‐Aviv Sourasky Medical Center Tel‐Aviv Israel; ^2^ Pediatric Dermatology Clinic Dana‐Dwek Children's Hospital, Tel‐Aviv Sourasky Medical Center Tel‐Aviv Israel; ^3^ Sackler Faculty of Medicine Tel‐Aviv University Tel‐Aviv Israel; ^4^ Department of Dermatology, Hadassah University Hospital Faculty of Medicine, Hebrew University Jerusalem Israel; ^5^ Department of Pathology Tel‐Aviv Sourasky Medical Center Tel‐Aviv Israel

## Abstract

Generalized acquired dermatoses can seldom manifest more prominently or exclusively along the lines of Blaschko. Six individuals with segmental atopic dermatitis (AD) have been reported to date. We present three additional cases of segmental cutaneous manifestations superimposed on generalized AD, and review the relevant literature.

## Graphical abstract

Generalized acquired dermatoses can seldom manifest more prominently or exclusively along the lines of Blaschko. Six individuals with segmental atopic dermatitis (AD) have been reported to date. We present here three additional cases of segmental AD that support the existence of this rare and often treatment‐resistant form of AD.

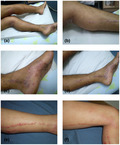

Atopic dermatitis (AD) is one of the most common skin diseases. Segmental manifestations are exceedingly rare in AD with only six cases reported to date.^1^, ^2^, ^3^ We describe three additional patients who manifested with distinctly pronounced linear lesions of AD on a background of a milder and generalized disease.

## Report

**Figure 1:**
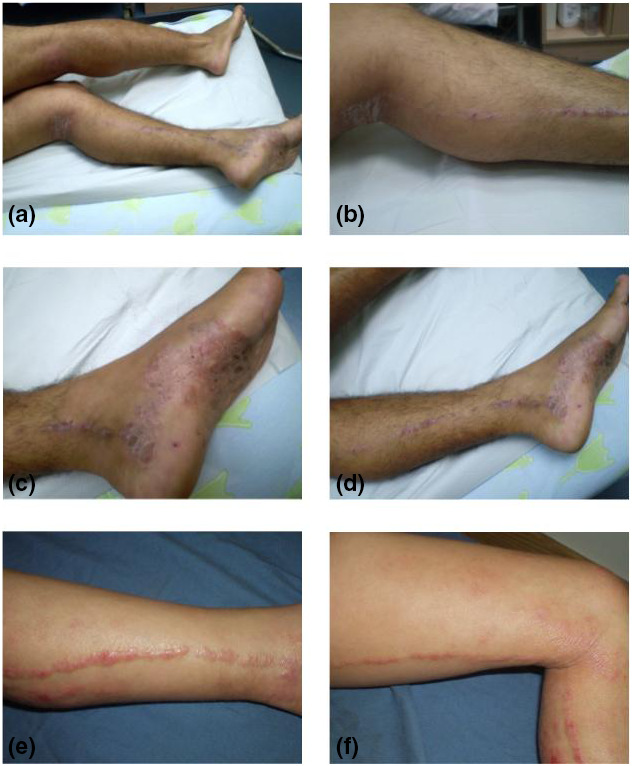
(a–d) Patient 1: linear rash on left popliteal fossa and medial aspect of the foot composed of papules and plaques covered with scales, crust and lichenification. (e,f) Patient 3: linear rash on the left thigh, leg and ankle composed of erythematous papules and plaques with an impressive rope‐like lichenification. [Colour figure can be viewed at wileyonlinelibrary.com]

**Figure 2:**
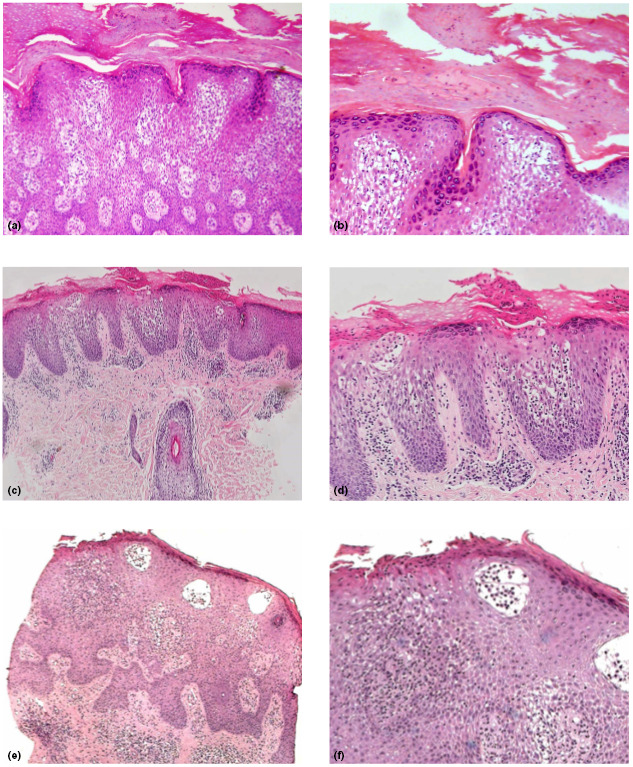
(a,b) Patient 1: hyperkeratosis with foci of parakeratosis, extensive spongiosis, preserved stratum granulosum and extensive perivascular infiltrates. (c,d) Patient 2: perivascular, spongiotic psoriasiform dermatitis with orthohyperkeratosis and focal parakeratosis, compatible with lichen simplex superimposed on atopic dermatitis. (e,f) Patient 3: irregular acanthosis, packed hyperkeratosis and parakeratosis, marked spongiosis, and perivascular and interstitial infiltrates. Haematoxylin and eosin, original magnification (a) × 20; (b) × 40; (c) × 10; (d) × 20; (e) × 10; (f) × 20. [Colour figure can be viewed at wileyonlinelibrary.com]

Patient 1, a 31‐year‐old man with no relevant family history, presented with a 2‐year history of a pruritic rash overlying extensive parts of his body. Physical examination of the patient revealed well‐demarcated, red–brown scaly papules and plaques with lichenification, involving the scalp, trunk, axillae, inner aspect of right elbow and popliteal fossae, suggestive of a clinical diagnosis of AD. In addition, erythematous papules and plaques covered with scales, crust and lichenification, were noted to extend linearly from the left popliteal fossa to the medial aspect of the foot (Fig. 1a–d). Laboratory tests were unremarkable except for elevated IgE level. Histopathological examination of a skin biopsy obtained from the linear lesion at the medial border of the left foot revealed acral skin with hyperkeratosis and foci of parakeratosis, extensive spongiosis, preserved stratum granulosum and extensive perivascular infiltrates (Fig. 2a,b), compatible histologically with AD. The presence of a linear complex of lesions combining clinical and histological features suggestive of AD, on the background of generalized AD and elevated IgE, suggested the diagnosis of superimposed Type 2 segmental AD. Improvement was noted after the use of topical corticosteroids however, the segmental rash was more resistant to treatment.

Patient 2 was a 6‐year‐old boy with AD since early childhood, who presented with an 18‐month history of an intensely pruritic linear rash, affecting the left shin and left knee. His father had asthma. Clinical examination of the child revealed a segmental lesion composed of hypopigmented plaques with lichenification, partly covered by mild scale (unfortunately, clinical photography was not taken). Histology from the right shin showed a perivascular, spongiotic psoriasiform dermatitis with orthohyperkeratosis and focal parakeratosis, compatible with lichen simplex superimposed on AD (Fig. 2c,d). The linear distribution of the rash on the background of generalized AD, the histological findings and the pruritic nature of the rash also suggested superimposed Type 2 segmental AD. Improvement was noted after the use of topical corticosteroids, pimecrolimus cream 1% and emollients.

Patient 3 was a 7‐year‐old boy, who was diagnosed with AD in early childhood, and was admitted to hospital due to severe exacerbation of his skin disease. Over the years, he had been treated with emollients and topical corticosteroids with mild to moderate improvement. At the age of 6 years, he had developed a pruritic linear lesion over his left thigh, leg and ankle, which was prominent during exacerbation of the atopic lesions and responded partially to topical treatment for AD. Physical examination revealed multiple eczematous plaques on the child's face, thorax and extremities, and on his left thigh, leg and ankle there was a linear lesion along the lines of Blaschko, composed of erythematous papules and plaques with an impressive rope‐like lichenification (Fig. 1e,f). Histology from the linear lesion demonstrated irregular acanthosis, packed hyperkeratosis and parakeratosis, marked spongiosis in the Malpighian layer, and perivascular and interstitial infiltrates composed of mononuclear cells and a few eosinophils in the upper dermal layer (Fig. 2e,f). Taken together, the clinical and pathological findings also pointed to the diagnosis of superimposed Type 2 segmental AD. The child was initially treated with coal tar 5% ointment and broad‐spectrum ultraviolet (UV)B phototherapy. After remission was achieved, he continued ambulatory phototherapy with narrowband UVB in combination with topical steroids and emollients.

A patterned distribution of skin lesions was first described by Blaschko in 1901,^4^ and has been reported not only in the context of monogenic diseases but also in a wide range of so‐called acquired conditions. These conditions include linear epidermal naevus with inflammatory linear verrucous epidermal naevus, lichen striatus (occasionally in association with AD^5^, ^6^), psoriasis, lichen planus, systemic lupus erythematosus, pemphigus vulgaris, vitiligo, graft‐versus‐host disease, granuloma annulare, erythema multiforme and drug eruption.^5^, ^6^, ^7^, ^8^, ^9^ Segmental manifestations of AD are exceedingly rare, with only six cases reported to date.^1^, ^2^, ^3^

This patterned distribution of skin lesions results from an early postzygotic event of loss of heterozygosity, resulting in hemizygosity or homozygosity of alleles predisposing to the disease.^1^, ^2^, ^3^, ^7^, ^8^, ^9^ In Type 1 mosaicism, the patterned distribution of the skin lesions is restricted to the lines of Blaschko, whereas in Type 2 mosaicism, severe segmental manifestations are superimposed on the general and milder form of a generalized disease in the same patient,^8^ as demonstrated in our three cases.

In conclusion, we present three cases of superimposed segmental AD, supporting the existence of this rare and often treatment‐resistant form of AD. Segmental forms of common disorders may be instrumental in the identification of critical elements in the pathogenesis of these conditions, eventually leading to targeted treatment as recently demonstrated.^10^ Somatic mutations may underlie a large array of both cutaneous and extracutaneous disorders, further emphasizing the importance of postzygotic genetic events to human pathologies.^11^

**Learning points**

- Acquired cutaneous conditions such as AD can manifest as segmental disorders, reflecting a postzygotic event leading to loss of heterozygosity.
- The presence of segmental AD on the background of milder generalized AD is suggestive of Type 2 mosaicism.
- Segmental forms of common disorders may be instrumental in the identification of critical elements in the pathogenesis of these conditions, eventually leading to targeted treatment.

## Conflict of interest

The authors declare that they have no conflicts of interest.

## Funding

None.

## Ethics statement

Ethics approval is not applicable. The patient has provided informed consent to publication of their case details and images.

## Data availability

Not applicable.
